# Neurobeachin regulates receptor downscaling at GABAergic inhibitory synapses in a protein kinase A-dependent manner

**DOI:** 10.1038/s42003-024-07294-z

**Published:** 2024-12-12

**Authors:** Felix P. Lützenkirchen, Yipeng Zhu, Hans M. Maric, Dominik S. Boeck, Kira V. Gromova, Matthias Kneussel

**Affiliations:** 1 Department of Molecular Neurogenetics, Center for Molecular Neurobiology, ZMNH, University Medical Center Hamburg-Eppendorf, Hamburg, Germany; 2 Rudolf Virchow Center, Center for Integrative and Translational Bioimaging, University of Würzburg, Würzburg, Germany; 3 Hamburg Center of Neuroscience, HCNS, University Medical Center Hamburg-Eppendorf, Hamburg, Germany

**Keywords:** Cellular neuroscience, Molecular neuroscience

## Abstract

GABAergic synapses critically modulate neuronal excitability, and plastic changes in inhibitory synaptic strength require reversible interactions between GABA_A_ receptors (GABA_A_Rs) and their postsynaptic anchor gephyrin. Inhibitory long-term potentiation (LTP) depends on the postsynaptic recruitment of gephyrin and GABA_A_Rs, whereas the neurotransmitter GABA can induce synaptic removal of GABA_A_Rs. However, the mechanisms and players underlying plastic adaptation of synaptic strength are incompletely understood. Here we show that neurobeachin (Nbea), a receptor trafficking protein, is a component of inhibitory synapses, interacts with gephyrin and regulates the downscaling of inhibitory synaptic transmission. We found that the recruitment of Nbea to GABAergic synapses is activity-dependent and that Nbea regulates GABA_A_R internalization in a protein kinase A (PKA)-dependent manner. In heterozygous neurons lacking one Nbea allele, re-expression of Nbea but not expression of a PKA binding-deficient Nbea mutant rescued the internalization of GABA_A_Rs. Our data suggest a mechanism by which Nbea mediates PKA anchoring at inhibitory postsynaptic sites to downregulate GABAergic transmission. They emphasize the importance of kinase positioning in the regulation of synaptic strength.

## Abstract (web summary)

Recruitment of Neurobeachin to GABAergic synapses facilitates the downscaling of inhibitory synaptic strength by regulating GABA_A_ receptor internalization in a PKA-dependent manner, emphasizing the role of Neurobeachin in synaptic plasticity.

## Introduction

The balance between excitation and inhibition is crucial for maintaining efficient information processing in the human brain^1^. In addition to the physiological changes underlying synaptic transmission and plasticity, disruption of this balance can lead to drastic pathophysiological and/or behavioral consequences, ranging from autism spectrum disorders (ASD) and other neuropsychiatric disorders to epileptic seizures^2^. GABA_A_ receptors (GABA_A_Rs) are the major types of inhibitory neurotransmitter receptors in the adult brain, responsible for the fast-synaptic response to γ-aminobutyric acid (GABA). The adaptive regulation of their cell surface expression through release, internalization, and recycling is a crucial factor for synaptic plasticity^3,4^.

To form functional inhibitory synapses, neuroligin-2 (NLGN2) accumulates in front of the presynaptic GABAergic terminals due to its interactions with neurexins, where it then forms a tripartite complex with the guanine nucleotide exchange factor collybistin and gephyrin^5,6^. Collybistin induces the formation of a multimeric gephyrin scaffold^6–11^, which in turn anchors GABA_A_Rs at the synapse^12–14^. NLGN2, gephyrin, and GABA_A_R subunits undergo post-translational modification by phosphorylation at different target sites that alter the clustering properties of receptors and signaling molecules to scale inhibitory synapse function^5,15–19^.

For synapse strengthening after the induction of inhibitory long-term potentiation (iLTP), Ca^2+^/calmodulin-dependent protein kinase II (CaMKII) mediates the recruitment of extrasynaptic gephyrin and the immobilization of GABA_A_Rs at inhibitory postsynaptic sites^20^. In contrast, prolonged exposure to the neurotransmitter GABA leads to a reduction in inhibitory synaptic currents through endocytosis of GABA_A_Rs^21,22^, a process thought to involve phosphorylation mediated by protein kinase A (PKA), which is known to alter a critical serine residue in α2 GABA_A_R subunits^23,24^. PKA also regulates the cell surface abundance of NLGN2 in a peptidylprolyl isomerase (PIN1)-dependent manner^19,25^ and PKA activity can be facilitated by A-kinase anchor proteins (AKAPs), such as protein 79/150, interacting with GABA_A_R-β subunits^26^. In general, the binding affinity of kinases to anchoring proteins appears to be decisive for the orientation of kinases in the vicinity of their substrates^27^, but the regulation of kinase positioning, its activity dependence, and its contribution of synaptic scaling is presently poorly understood.

Neurobeachin, also known as Nbea, is a neuron-specific protein that binds protein kinase A^28^. Nbea is involved in the trafficking and signaling of membrane receptors^29^ and depletion of Nbea disrupts the formation of excitatory synapses or impairs synaptic transmission at neuromuscular junctions^30–32^. Heterozygous Nbea mutants in patients and mice have been associated with neurological and neurodevelopmental disorders, including epilepsy or ASD, respectively^33–36^. Nbea contributes to synaptic receptor targeting via the biosynthetic secretory pathway^37^ and regulates recycling of glutamate receptors at excitatory synapses^38^. In contrast, little is known about whether and how Nbea is involved in the regulation of GABA_A_Rs at inhibitory postsynaptic sites.

In this study, we report that Nbea is recruited to GABAergic synapses. We found that synaptic activity-dependent protocols regulate the synaptic localization of GABA_A_Rs and Nbea in a reciprocal manner. Nbea binds to the gephyrin scaffold and anchors PKA at GABAergic postsynaptic positions. Remarkably, inhibition of PKA or overexpression of a neurobeachin deletion mutant lacking the PKA binding domain impairs internalization of GABA_A_Rs. Our data emphasize the necessity for anchor proteins in positioning kinases and identify Nbea as a component of the inhibitory synapse that regulates downscaling of GABA_A_Rs.

## Results

### Nbea is localized at activated GABAergic synapses

**Fig. 1:**
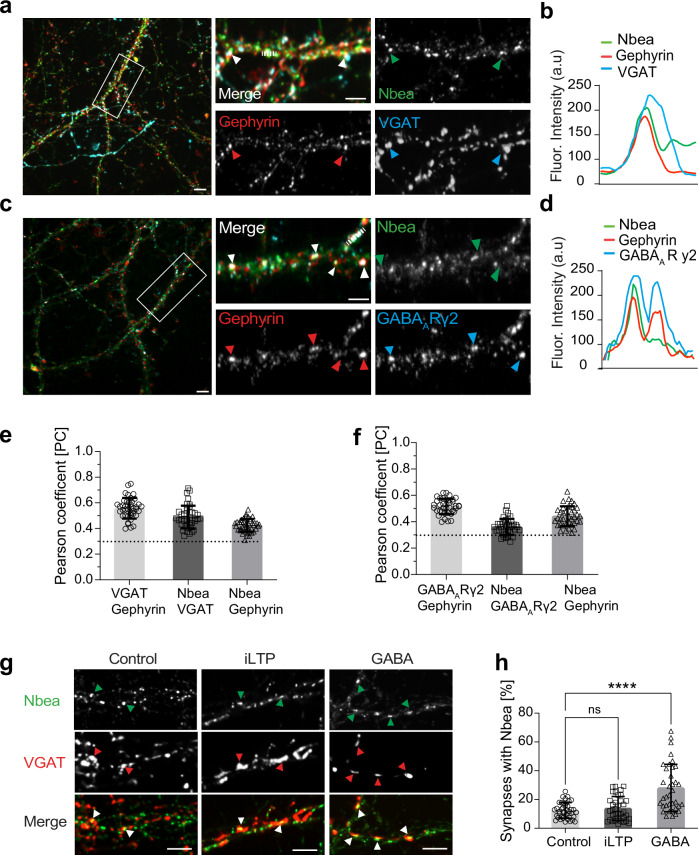
Neurobeachin is recruited to GABAergic synapses in an activity-dependent manner. **a** Hippocampal neurons immunostained for endogenous NBEA (green), endogenous gephyrin (red), and endogenous VGAT (blue). Arrows depict examples of colocalized signals. **b** Line scan depicting the overlapping fluorescent signal intensities (arbitrary units, a.u.) along the dashed line in (**a**). **c** Hippocampal neurons immunostained for endogenous NBEA (green), endogenous gephyrin (red) and endogenous GABA_A_Rγ2 (blue). Arrows depict examples of colocalized signals. **d** Line scan depicting the overlapping fluorescence signal intensities (arbitrary units, a.u.) along the dashed line shown in (**c**). **e** Pearson correlation coefficient measured between the colocalization of VGAT/gephyrin, Nbea/VGAT, and Nbea/gephyrin, and plotted for 3 independent experiments, *n* = 41 cells. **f** Pearson correlation coefficient measured between the colocalization of GABA_A_Rγ2/gephyrin, Nbea/GABA_A_Rγ2, and Nbea/gephyrin, and plotted for 3 independent experiments, *n* = 38 cells. **g** Hippocampal neurons treated with a CNQX/NMDA protocol to induce iLTP or GABA co-immunostained against endogenous NBEA (green), endogenous VGAT (red). Arrows depict examples of colocalized signals. **h** Percentage of NBEA-positive synapses determined by colocalization of NBEA, VGAT, and gephyrin comparing the different conditions in (**g**) (Control vs. iLTP *p* = 0.8074; Control vs. GABA ****p < 0.0001). 3 independent experiments, *n* = 36 cells. Scale bars 2 µm (**a**, **f** and **c**, upper) and 1 µm (**c**, lower). Data expressed as mean ± SD. Statistical significance determined using One-way ANOVA (**h**).

Since Nbea has been functionally associated with the clustering and turnover of synaptic AMPA- and NMDA-receptors at excitatory synapses, respectively^32,38^, we wondered whether it would be generally detectable at GABAergic synapses and whether its subcellular localization might be activity-dependent. Immunostaining with antibodies specific for Nbea and the pre- and postsynaptic markers VGAT and gephyrin, respectively, identified a series of triple-colocalized puncta at inhibitory synaptic sites (Fig. 1a, arrows). Despite the wide distribution of Nbea as a trafficking factor, a subset of endogenous Nbea signals exhibited overlapping fluorescent intensities with both inhibitory synaptic markers (Fig. 1b). Accordingly, endogenous Nbea was detected at individual gephyrin/GABA_A_R γ2 double-positive clusters (Fig. 1c, arrows, Fig. 1d, left peak), while other gephyrin/GABA_A_R γ2 puncta were found to be Nbea-negative (Fig. 1d, right peak). Quantification with Pearson correlation coefficients revealed a moderate colocalization of all proteins with Nbea (Fig. 1e, f). suggesting that Nbea, which is a widely distributed trafficking factor, localizes to inhibitory synapses only under certain conditions. Chemical protocols for the induction of inhibitory LTP (iLTP) recruit gephyrin and GABA_A_Rs to inhibitory postsynaptic sites^20^, while abundant GABA induces downregulation of inhibitory synaptic transmission via internalization of GABA_A_Rs^21,22^. To test whether these effects can be mimicked in cultured hippocampal neurons, we chemically induced iLTP or administered GABA followed by triple-immunostaining. As expected, induction of iLTP resulted in a significant increase in GABA_A_R clusters (Supplementary Fig. 1a, b, middle) as well as in the synaptic area of inhibitory synapses (Supplementary Fig. 1a, c, middle), as evidenced by triple colocalization of presynaptic VGAT, postsynaptic gephyrin, and the GABA_A_R γ2 subunit. In contrast, administration of 100 µM GABA significantly reduced the area of GABA_A_R clusters (Supplementary Fig. 1a, b, right) as well as the size of inhibitory synapses (Supplementary Fig. 1a, c, right), compared to unstimulated conditions (Supplementary Fig. 1a–c, left). We therefore wondered whether the subcellular localization of Nbea could also respond to the activity changes induced by these protocols. While induction of iLTP had no effect on Nbea localization (Fig. 1g, h, middle), application of GABA significantly increased the number of VGAT-positive sites that contained Nbea signals (Fig. 1g, h, right). These data show that Nbea targets VGAT-positive sites at elevated GABA levels, which remove GABA_A_R γ2-containing receptors from inhibitory synapses (Supplementary Fig. 1a, b), suggesting that Nbea may participate in the redistribution of GABA_A_Rs.

### Nbea binds to the gephyrin/GABA_A_R complex

**Fig. 2:**
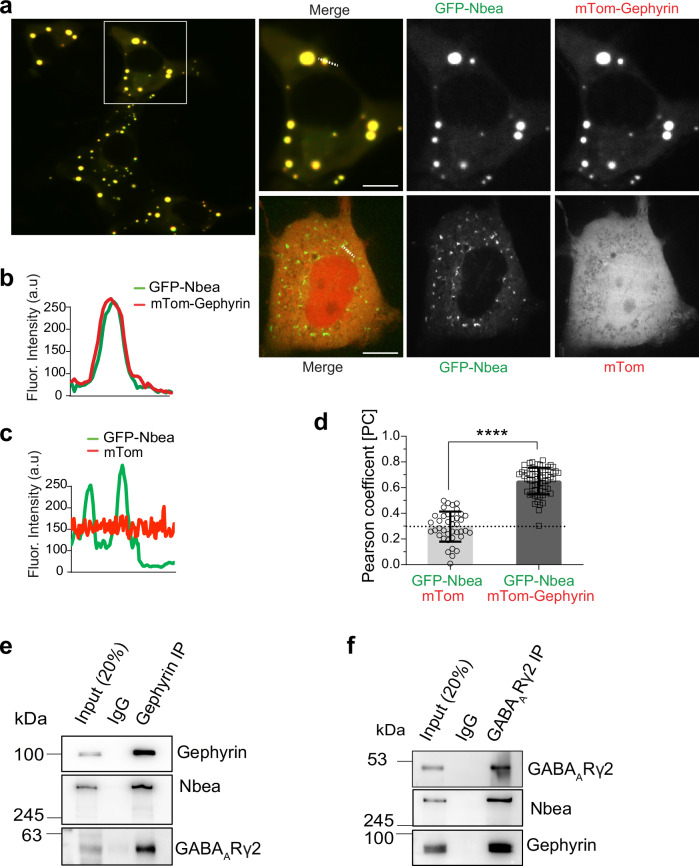
Nbea interacts with the gephyrin-GABA_A_R complex. **a** COS-7 cells expressing GFP-Nbea (green) with either mTom-gephyrin or mTom (red). 3 independent experiments. **b**, **c** Line scans depicting the overlapping fluorescent signal intensities (arbitrary units, a.u.) above the white lines in (**a**). **d** Pearson correlation coefficient measured between the colocalization of GFP-Nbea/mTom and GFP-Nbea/mTom-Gephyrin and compared (****p < 0.0001) for 3 independent experiments, *n* > 40 cells. **e** Immunoprecipitation of gephyrin from adult mouse cortical lysate. Co-immunoprecipitation of Nbea and GABA_A_Rγ2. 3 independent experiments. **f** Immunoprecipitation of GABA_A_Rγ2 from adult mouse cortical lysate. Co-immunoprecipitation of Nbea and Gephyrin. 3 independent experiments. Scale bar: 10 µm in (**a**). Data expressed as mean ± SD. Statistical significance determined using Student’s t-test (**d**).

In order to downregulate GABA_A_Rs (compare with Supplementary Fig. 1a, b, right) the receptors would have to uncouple from the subsynaptic gephyrin scaffold. Assuming that Nbea might be involved in this process, we asked whether Nbea interacts with gephyrin. Gephyrin is known to accumulate in the cytoplasm of immortalized kidney cells after heterologous expression^39^. To determine whether Nbea might be capable of associating with gephyrin in a non-neuronal context, we overexpressed GFP-Nbea together with mTom-gephyrin in Cos-7 fibroblast-like cells. Indeed, GFP-Nbea was strongly recruited into cytoplasmic gephyrin clusters (Fig. 2a, upper, 2b) but did not colocalize with mTom alone (Fig. 2a, c). This result was confirmed by a strong increase in the Pearson correlation coefficient compared to control conditions (Fig. 2d). Consistent with the colocalization of these factors, co-immunoprecipitation experiments (co-IPs) revealed a protein complex consisting of Nbea, GABA_A_Rγ2-containing receptors, and gephyrin. In these experiments, gephyrin antibodies precipitated endogenous gephyrin and led to co-precipitation of Nbea and GABA_A_R γ2-containing receptors (Fig. 2e). Conversely, GABA_A_R γ2-specific antibodies precipitated the receptor from mouse cortical lysates and led to co-precipitation of Nbea and gephyrin (Fig. 2f). In addition, stringently washed, co-immunoprecipitated GFP-Nbea also pulled down purified gephyrin (Supplementary Fig. 2), suggesting the formation of a strong complex between Nbea and gephyrin.

**Fig. 3:**
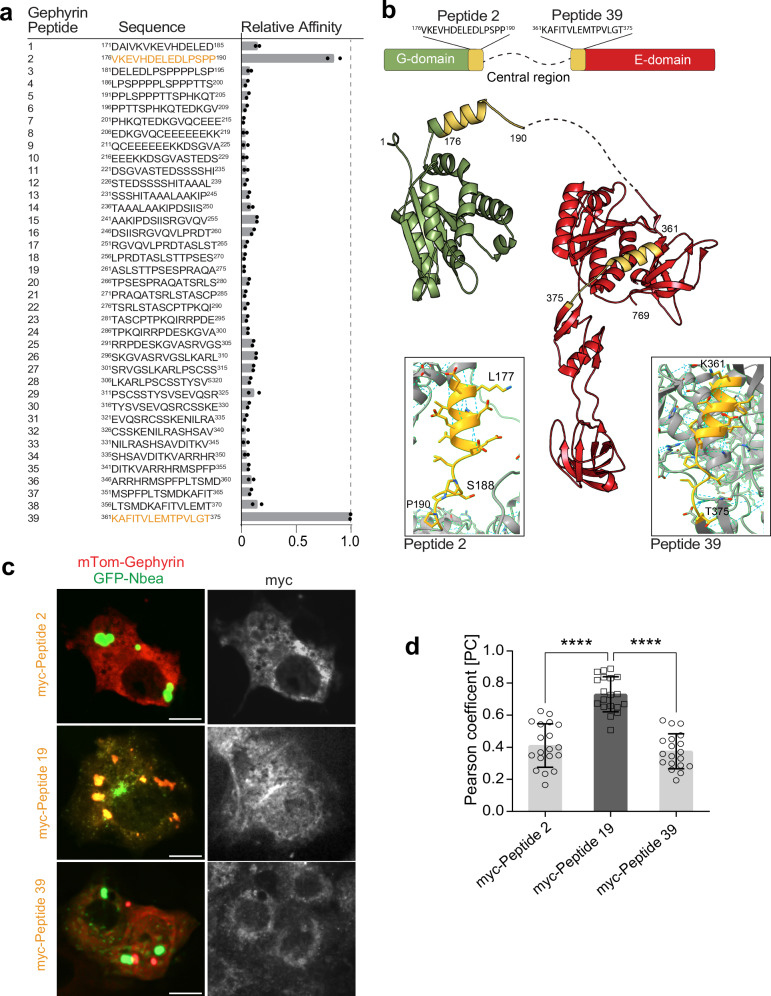
Two sequences in the gephyrin C- and E-domains a crucial for Nbea binding. **a** Relative binding intensity of Nbea to different peptides of gephyrin (GPHN-1 isoform), *n* = 2 independent experiments. **b** Domain structure and ribbon diagram of gephyrin (AF-Q9NQX3-F1) with a close up of peptides 2 and 39. **c** COS-7 cells expressing GFP-Nbea (green) and mTom-gephyrin (red) with either myc-Peptide 2, myc-Peptide 19 or myc-Peptide 39. **d** Pearson correlation coefficient comparing the colocalization of mTom-gephyrin and GFP-Nbea in (**c**) (Peptide 2 vs. Peptide 19 ****p < 0.001; Peptide 19 vs. Peptide 39 ****p < 0.001); 3 independent experiments, *n* = 19 cells; Scale bar: 10 µm (**c**). Data are expressed as mean ± SD. Statistical significance determined using One-way ANOVA.

To map critical interaction sites mediating the association of Nbea with gephyrin, we used peptide arrays with immobilized gephyrin peptides covering the central and putatively intrinsically disordered linker region (residues 171–375) between the N-terminal G-domain and C-terminal E-domain. Array incubation with whole brain lysate and probing for Nbea binding identified two gephyrin peptides No. 2 ^176^VKEVHDELEDLPSPP^190^ and No. 39 ^361^KAFITVLEMTPVLGT^375^ as mediators of the Nbea interactions (Fig. 3a and Supplementary Fig. 3a, b). Other peptides, such as gephyrin peptide No. 19 ^261^ASLSTTPSESPRAQA^275^, did not appear to be involved in complex formation with Nbea. Moreover, expression of peptides No. 2 or No. 39 in COS-7 cells that overexpressed mTom-gephyrin together with GFP-Nbea, was able to interfere with complex formation (Fig. 3c, bottom and top, 3 d), whereas the unrelated peptide No. 19 failed to do so (Fig. 3c, middle, 3 d). Overall, our data show that Nbea is associated with gephyrin upon heterologous expression and this complex is detectable in brain lysate. This suggests that the activity-dependent localization of Nbea at GABAergic postsynaptic sites is accomplished by a complex formation of Nbea and gephyrin.

### Depletion of Nbea impairs GABA_A_R internalization

**Fig. 4:**
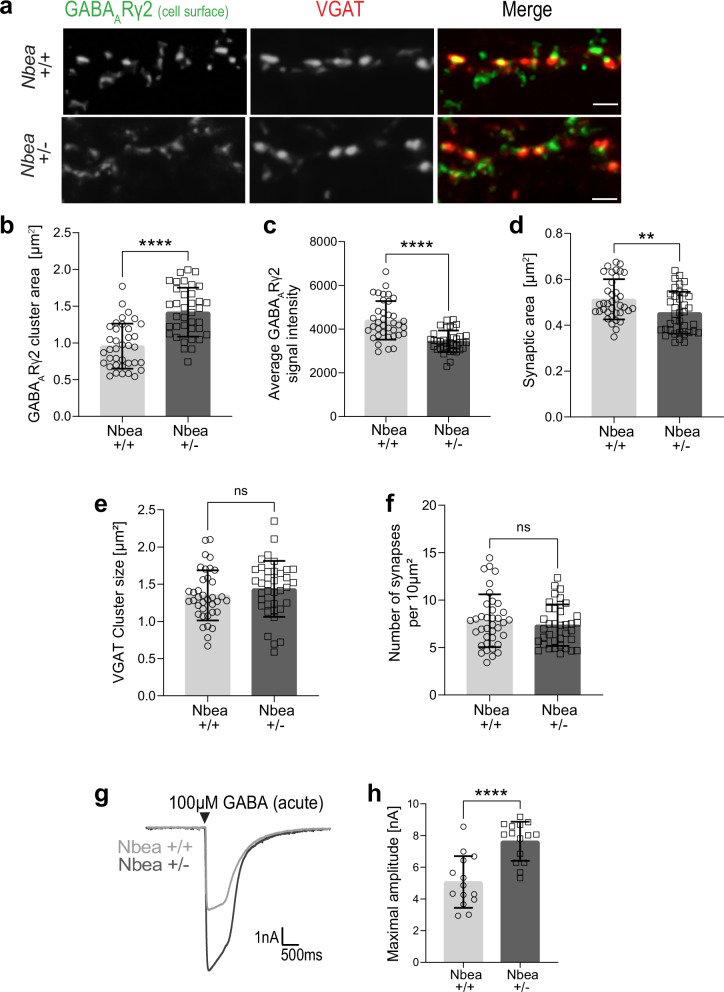
Nbea participates in the regulation of GABA_A_R surface levels. **a** Cultured hippocampal neurons from *Nbea* +/+ and *Nbea* +/− mice immunostained for endogenous GABA_A_Rγ2 at the cell surface (green, live cell staining protocol), and endogenous VGAT (red). **b** Average GABA_A_Rγ2 cluster area (µm^2^) compared between *Nbea* +/+ and *Nbea* +/− neurons in (**a**) (****p < 0.0001). 3 independent experiments, *n* = 37 cells. **c** Average GABA_A_Rγ2 signal intensity compared between *Nbea* +/+ and *Nbea* +/− neurons in (**a**) (****p < 0.0001). 3 independent experiments, *n* = 37 cells. **d** Synaptic area define**d** as GABA_A_Rγ2 and VGAT double-positive areas from *Nbea* +/+ and *Nbea* +/− neurons in (**a**) (**p = 0.0081).3 independent experiments, *n* = 37 cells. **e** Average VGAT clust**e**r size compared between *Nbea* +/+ and *Nbea* +/− neurons in (**a**) (*p* = 0.2995). 3 independent experiments, *n* = 37 cells. **f** Number of synapses per 10 µm^2^ counted in *Nbea* +/+ and *Nbea* +/− neurons in (**a**) (*p* = 0.4278). 3 independent experiments, *n* = 37 cells. **g** Representative responses to bath applied GABA (evoked IPSCs) from DIV 14–18 cultured hippocampal neurons of *Nbea* +/+ and *Nbea* +/− animals. **h** Maximal amplitude (nA) from (**c**) compared between *Nbea* +/+ and *Nbea* +/− neurons (***p < 0.001). 3 independent experiments, *n* = 14 cells. Scale bars (**a**) 2 µm. Data are represented as mean ± SD. Statistical significance determined using unpaired Student’s t-test (**b**–**e**, **h**) and Mann-Whitney U test (**f**).

**Fig. 5:**
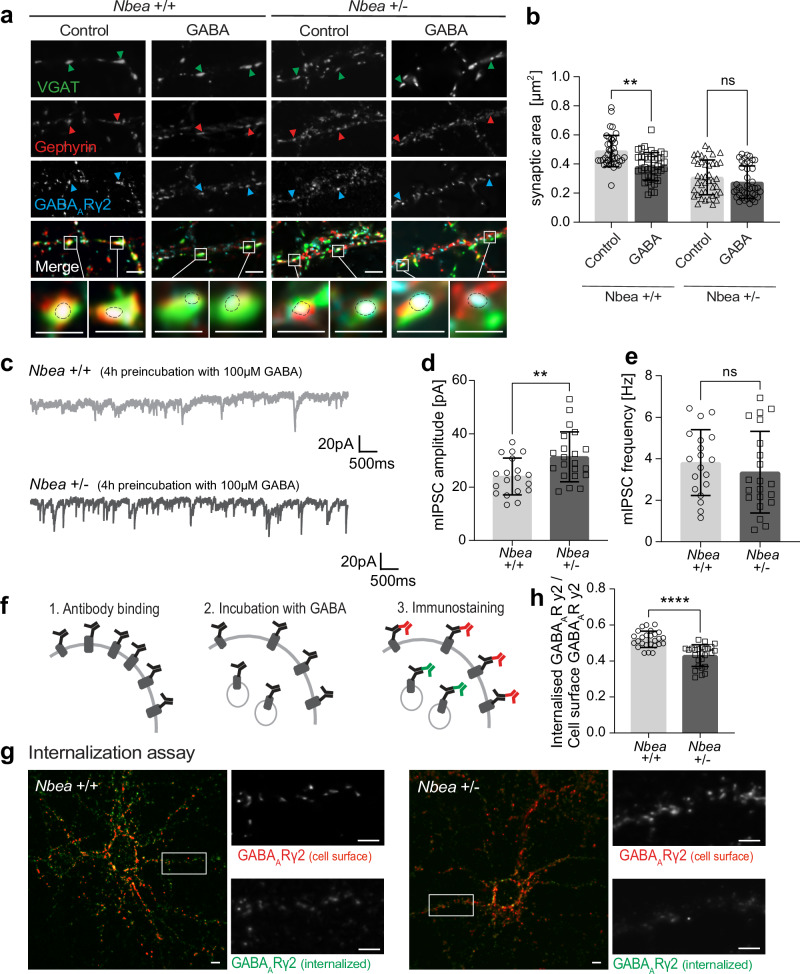
Nbea participates in the regulation of GABA_A_R surface levels. **a** Hippocampal neurons from *Nbea* +/+ and *Nbea* +/- mice immunostained for endogenous VGAT (green), endogenous gephyrin (red) and endogenous GABA_A_Rγ2 (blue). Arrows depict examples of colocalized signals. **b** Synaptic area (µm^2^) of triple-colocalized puncta in (**a**) compared between control and GABA (*Nbea* +/+ ***p = 0.00022, *Nbea* +/- *p* = 0.4736). 4 independent experiments, *n* = 41 cells. **c** mIPSCs recorded in hippocampal neurons from *Nbea* +/+ and *Nbea* +/- mice upon pre-stimulation with 100 µM GABA for 4 h. **d** Average mIPSCs amplitude from (**c**) in pA compared between *Nbea* +/+ and *Nbea* +/- neurons (***p = 0.0077). 3 independent experiments *Nbea* +/+ *n* = 19 cells, *Nbea* +/- *n* = 21 cells. **e** mIPSC frequency from (**c**) in Hz compared between *Nbea* +/+ and *Nbea* +/- neurons (*p* = 0.4253). 3 independent experiments *Nbea* +/+ *n* = 19 cells, *Nbea* +/- *n* = 21 cells. **f** Schematic representation of the ínternalization assay. (1) Labeling with primary antibody prior to internalization, (2) incubation with 100 µM GABA to induce internalization, (3) consecutive immunostaining of surface receptors against internalized receptors using secondary antibodies. **g** Internalization assay using hippocampal neurons from *Nbea* +/+ and *Nbea* +/- mice. Detection of internalized GABA_A_Rγ2-containing receptors (green) versus cell surface GABA_A_Rγ2-containing receptors (red). **h** Intensity of internalized GABA_A_Rγ2-containing receptors relative to the number of cell surface receptors from *Nbea* +/+ (*n* = 26 cells) or *Nbea* +/- neurons (*n* = 26 cells) (****p < 0.0001). 3 independent experiments. Data expressed as the mean ± SD. Scale bars: 2 µm. Statistical significance determined using unpaired Student’s t-test (**d**, **e**, **h**), or one-way ANOVA (**b**).

Since the gephyrin scaffold regulates GABA_A_R cell surface dynamics^39^, we considered it possible that Nbea plays a role in these processes. To test this hypothesis, we used hippocampal neurons derived from heterozygous *Nbea* knockout mice (*Nbea* +/−) that are characterized by a reduced Nbea protein expression. A complete loss of the *Nbea* gene disrupts synapse formation^31^, while *Nbea* heterozygosity maintains normal synaptic transmission^32,40^. Our analysis of the GABA_A_R γ2-containing receptors on the cell surface of *Nbea* +/− neurons showed a significant increase in the receptor cluster area (Fig. 4a, b), accompanied by a decrease in the average intensity of the individual clusters (Fig. 4a, c). The synaptic area, i.e. the pre- and postsynaptic apposition, was also significantly reduced in *Nbea* +/− neurons (Fig. 4a, d). At the same time, we could not detect any difference in the size of VGAT clusters or the number of synapses per se (Fig. 4a, e, f). Taken together, this suggests that reduced Nbea levels cause an increased dispersion of GABA_A_Rs shifting them to extrasynaptic positions. Electrophysiological recordings of IPSCs evoked by a relatively high amount of GABA (100 µM) resulted in significantly increased amplitudes, suggesting an increase of GABA_A_Rs in heterozygous Nbea +/− neurons, presumably at extrasynaptic plasma membrane regions (Fig. 4g, h). To check how this might affect synaptic plasticity, we repeated the GABA stimulation protocol (3 min GABA/15 min recovery), which had previously induced a reduction of GABA_A_Rs (see Supplementary Fig. 1a, b) and compared possible effects between wild-type (+/+) and Nbea +/− neurons (Fig. 5a, b). Of note, reduced Nbea levels (*Nbea* +/− neurons) abolished the ability of GABA stimulation to reduce the size of inhibitory synapses, as determined by quantifying VGAT/gephyrin/GABA_A_Rγ2 triply colocalized areas (Fig. 5a, b). In parallel, Nbea heterozygosity also affected inhibitory transmission, such as the amplitude but not the frequency of mIPSCs after chronic (4 h) GABA stimulation (Fig. 5c–e). Considering that *Nbea* +/− conditions do not affect inhibitory transmission per se or prior to treatment with GABA^32^, this supports the notion that Nbea may be required for GABA_A_R synaptic downscaling. To test this possibility, we performed antibody-feeding-based internalization assays to distinguish cell surface receptors (Fig. 5f, red) from receptors that had been internalized (Fig. 5f, green). In this assay, reduced levels of Nbea (*Nbea* +/− neurons) significantly decreased the ratio of internalized to plasma membrane receptors (Fig. 5g, h), thus supporting the concept that Nbea acts as a critical factor for the downscaling of GABA_A_Rs from inhibitory postsynaptic sites via internalization.

### Protein kinase A activity is essential for the downregulation of synaptic GABA_A_Rs

**Fig. 6:**
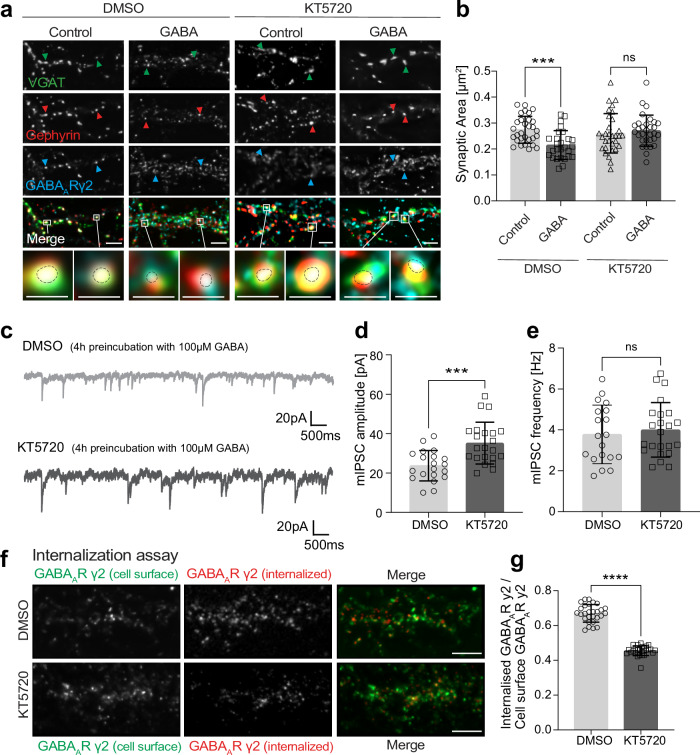
PKA activity mediates downscaling of GABAergic synapses. **a** Primary hippocampal neurons from *Nbea* +/+ mice stimulated with GABA upon DMSO or PKA-inhibitor KT5720 treatment, labeled for endogenous VGAT (green), endogenous gephyrin (red), and surface GABA_A_Rγ2 (blue) using specific antibodies. Arrows depict examples of colocalized signals. **b** Synaptic area (µm^2^) of triple-colocalized puncta in (**a**) compared between control and GABA (DMSO ***p = 0.0009; KT5720 *p* = 0.7661). 3 independent experiments; DMSO (control: *n* = 31 cells; GABA: *n* = 31 cells) or PKA-inhibitor KT5720 (control: *n* = 31 cells; GABA: *n* = 28 cells). **c** mIPSCs recorded in hippocampal neurons at DIV 12–14 treated with DMSO or PKA-inhibitor KT5720 after pre-stimulation with 100 µM GABA for 4 h. **d** Average mIPSCs amplitude from (**c**) in pA comparing neurons treated with DMSO or PKA-inhibitor KT5720 (***p = 0.0003). 3 independent experiments DMSO *n* = 20 cells, KT5720 *n* = 23 cells. **e** mIPSC frequency from (**c**) in Hz comparing *Nbea* +/+ and *Nbea* +/- neurons (*p* = 0.6097). 3 independent experiments DMSO *n* = 20 cells, KT5720 *n* = 23 cells. **f** Internalization assay in cultured hippocampal neurons conducted during treatment with DMSO (control) or PKA-inhibitor KT5720 (1 µM) and stained for cell surface GABA_A_ Rγ2 (green) and internalized GABA_A_Rγ2 (red). **g** Intensity of internalized GABA_A_Rγ2 receptors, quantified relative to the number of surface receptors treated either with DMSO or PKA-inhibitor KT5720 (****p < 0.0001) 3 independent experiments, *n* = 28 neurons. Scale bars (**a**, **f**): 2 µm. Data are represented as mean ± SD. Statistical significance determined using One-way ANOVA (**b**) or unpaired Student’s t-test (**d**, **e**, **g**).

Protein kinase A (PKA) plays an important role in the regulation of synaptic plasticity^41,42^ and Nbea binds the type II regulatory subunit of protein kinase A (PKA RII)^28^. To investigate a possible contribution of PKA to GABA-induced GABA_A_R downregulation at inhibitory synapses (see Supplementary Fig. 1a–c), we combined GABA stimulation with PKA inhibition by KT5720. While GABA administration reduced the synaptic area of triply colocalized VGAT/gephyrin/GABA_A_Rγ2 puncta under DMSO conditions, this effect was abolished in the presence of the PKA inhibitor KT5720 (Fig. 6a, b, compare with Fig. 5a, b). Likewise, increased IPSC amplitudes without changes in their frequency were detected upon chronic (4 h) stimulation with GABA in the presence of PKA inhibitor KT5720 (Fig. 6c–e, compare with Fig. 5c–e), suggesting that PKA crucially contributes to the downscaling of receptors at inhibitory postsynaptic sites. We therefore asked whether PKA might also be required for internalization of GABA_A_Rs from the neuronal cell surface and combined the previously used receptor internalization assay with PKA inhibition by KT5720. Under these conditions, the ratio of internalized to plasma membrane receptors was reduced in a similar manner as in heterozygous* Nbea* +/− neurons (Fig. 6f, g compare with Fig. 5f–h), confirming that internalization of GABA_A_R requires functional PKA, which in turn may depend on synaptic recruitment of PKA by Nbea.

### PKA binding to Nbea is crucial to rescuing Nbea loss of function

**Fig. 7:**
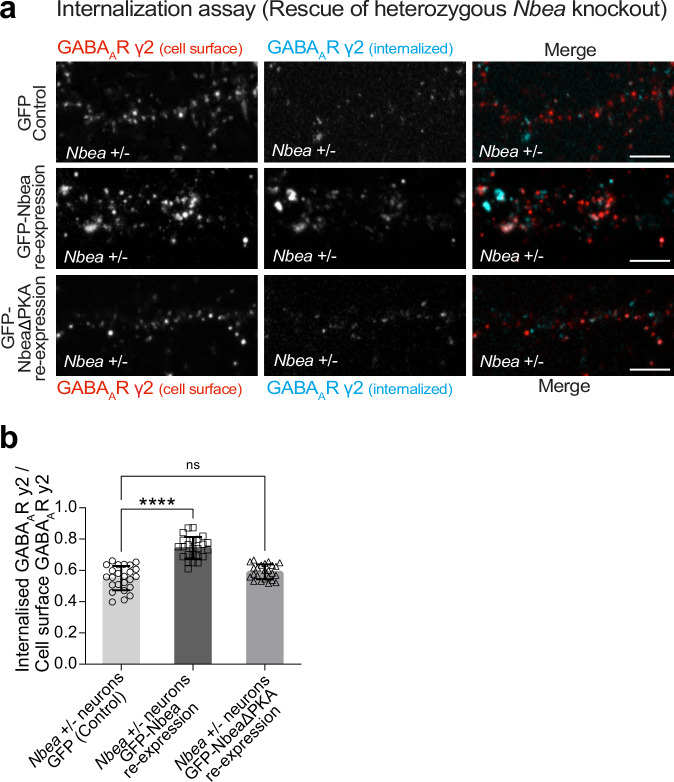
PKA anchoring by Nbea is crucial to rescuing Nbea loss of function. **a** Hippocampal neurons from *Nbea* +/- mice transfected with either GFP, GFP-Nbea-WT or GFP-NbeaΔPKA, incubated with primary antibodies specific for GABA_A_Rγ2 and immunostained with secondary antibodies to distinguish cell surface GABA_A_Rγ2 (red) and internalized GABA_A_Rγ2 (blue). **b** Intensity of internalized GABA_A_Rγ2-containing receptors quantified relative to the number of cell surface receptors upon the different conditions. (Control vs. WT ****p < 0.0001; Control vs. NbeaΔPKA *p* = 0.0741). 3 independent experiments (Control *n* = 23 cells; Nbea WT *n* = 26 cells; NbeaΔPKA *n* = 24 cells). Scale bars in a: 2 µm. Data represented as mean ± SD. Statistical significance determined using One-way ANOVA (**b**).

Finally, we asked whether Nbea could promote PKA activity at GABAergic synapses. If so, re-expression of Nbea in *Nbea* +/− knockout neurons should normalize the internalization deficit of GABA_A_Rs (see Fig. 5g, h) and lead to increased internalization compared to control conditions, whereas this should not be the case in an Nbea mutant lacking the PKA-binding domain. First, we used co-IP to confirm that deletion of the PKA binding site in Nbea indeed reduces its interaction with PKA (Supplementary Fig. 4a, b). We then repeated the neuronal receptor internalization assay after expression of either the control vector, Nbea or the Nbea deletion mutant (NbeaΔPKA) on the genetic background of *Nbea* +/− neurons. In this rescue experiment, transfected neurons were identified by simultaneous GFP expression. Based on our previous results showing that reduced levels of Nbea (*Nbea* +/−) impair the internalization of GABA_A_Rs (Fig. 5g, h), we expected opposite effects when Nbea was re-expressed. Indeed, the ratio of internalized to plasma membrane receptors was found to be significantly increased in neurons that re-expressed GFP-Nbea in *Nbea* +/− neurons (Fig. 7a, b, middle). However, this effect was lost in neurons overexpressing the GFP-Nbea mutant lacking the PKA-binding domain (Fig. 7a, b right), suggesting that Nbea-PKA binding is a critical determinant in the Nbea-mediated internalization of GABA_A_Rs.

## Discussion

Tight regulation of postsynaptic GABA_A_Rs is essential for the maintenance of proper synaptic function, and GABA_A_R dysregulation is associated with severe pathophysiological consequences. The data of this study highlight a role of Nbea-PKA binding in regulating agonist-induced GABA_A_R synaptic downscaling, showing that (i) stimulation with GABA recruits Nbea to inhibitory synapses, (ii) Nbea interacts with the GABA_A_R/gephyrin complex, (iii) depletion of Nbea impairs GABA_A_R internalization, (iv) PKA activity is required for the downregulation of synaptic GABA_A_Rs and (v) an Nbea mutant lacking PKA-binding cannot rescue Nbea loss-of-function conditions.

Nbea is a tubulovesicular protein known to be involved in the biosynthetic delivery of neurotransmitter receptors^32,37^, and is critical for spine formation and synaptogenesis^31^. Nbea binds to and colocalizes with inhibitory glycine receptors in the spinal cord^43^, but whether Nbea is associated with GABAergic synapses in the brain has remained unknown.

We report that Nbea is recruited to GABAergic synapses in an activity-dependent manner. At inhibitory synapses, interactions of Nbea with the gephyrin/GABAAR complex (Fig. 2) might occur via two distinct gephyrin binding sites (Fig. 3). Interestingly, one of them contains serine 188, a potential phosphorylation site previously associated with the binding of the chaperone Pin1^44^, and proposed to control neuroligin2/gephyrin interactions^25^, suggesting that Nbea may regulate this complex. Since posttranslational modifications on gephyrin influence its clustering^45^, it is possible that Nbea-gephyrin interactions could also be phospho-dependent. Mechanistically, our data suggest that Nbea recruits PKA to gephyrin/GABA_A_R complexes, as efficient internalization of GABA_A_Rs requires (i) PKA activity (Fig. 6) and Nbea-PKA binding (Fig. 7). It has been reported that activation of PKA requires elevated levels of cyclic adenosine monophosphate (cAMP)^46^. In response to GABA stimuli, cAMP could arise as a consequence of GABA_B_ receptor-mediated stimulation of adenylyl cyclase activity^47,48^, thereby inducing Nbea-dependent downscaling of GABA_A_Rs. However, PKA activity could also be regulated by other mechanisms, such as sustained activation of GABA and the resulting hyperpolarization of the neuron.

Currently, the substrate of PKA in this context is not known, but there is evidence that individual GABA_A_R subunits are phosphorylated by PKA and that their surface expression is regulated in a PKA-dependent manner^4,23,24,49^. Similarly, serine 303 of gephyrin is phosphorylated by PKA, although this is likely required for an activity-dependent increase in gephyrin clustering^50,51^. Interestingly, PKA-mediated phosphorylation of the synaptic adhesion molecule NLGN2 leads to a reduction of NLGN2 cell surface levels and synaptic destabilization, also inducing a secondary loss of GABA_A_Rs from inhibitory synapses^19^. We therefore conclude that PKA-mediated phosphorylation may relax gephyrin-GABA_A_R interactions, allowing the receptor to enter clathrin-coated pits, an AP2-dependent mechanism for the internalization of membrane proteins at inhibitory synapses^4,22,52,53^.

On the circuit level, previous reports linked GABA_A_R clustering with cognitive abilities in mice^54^ and over-inhibition of the hippocampal CA3 region correlates with a reduction in spatial memory in different studies^55–58^. Meanwhile, disruption of one *Nbea* allele through a translocation induces the onset of epileptic seizures and ASD^33,34^, and *Nbea* haploinsufficiency in mice induces autism-like behaviors, including a reduction in spatial memory^35,36^. A loss of both *Nbea* alleles (-/-) reduces the amplitude as well as the frequency of postsynaptic potentials due to a deficiency in synapse formation^32^. In contrast, basal synaptic transmission of excitatory and inhibitory synapses was found to be unchanged in heterozygous *Nbea*+/− mutants^32,40^, suggesting normal synapse numbers. Consistent with this view, our data show no differences in the number of GABA-ergic synapses, but impaired removal of synaptic GABA_A_ receptors following their activation (Fig. 4d). This suggests impaired synaptic downscaling of GABAergic synapses rather than a deficiency in synaptogenesis in the heterozygous *Nbea* +/− mutants. Based on observations within other mutant mouse lines, we assume that an accumulation of GABA_A_R at the cell surface followed by an enhanced efficacy of inhibitory synapses may contribute to some of the cognitive effects seen upon depletion of Nbea. However, further studies are needed to determine the exact cause of the behavioral abnormalities and their clinical relevance.

In summary, the data of the present study demonstrate an integral role of Nbea at inhibitory GABAergic synapses and highlight a PKA-dependent mechanism to internalize GABA_A_Rs in GABA-induced downscaling of synaptic transmission. They verify and extend previous models of PKA function at inhibitory GABAergic synapses^19^ by (i) confirming that PKA induces a reduction of synaptic GABA_A_Rs and (ii) adding Nbea as a critical determinant of PKA recruitment to the postsynaptic NLGN2/gephyrin/GABA_A_R complex. Furthermore, they highlight synaptic kinase positioning as an active, activity-dependent mechanism for bringing these cytosolic diffusible enzymes into proximity with their substrates.

## Methods

### Animals

The generation of Nbea-knockout (−/−) mice is described in detail elsewhere^59^. Both wild-type C57/BL/6 and Nbea +/− C57/BL/6 mice were housed and bred in the animal facility of the University Medical Center Hamburg-Eppendorf (UKE). The animals were housed in groups (2–5 mice per cage) and kept in an enriched environment on a 12-hour light/dark cycle, with food and water available ad *libitum*. The temperature (22 ± 1 °C) and humidity (50 ± 5%) in the animal facility were kept constant. For all experiments, 2- to 4-month-old mice of both sexes were used. All procedures were monitored and performed in accordance with German legislation and the guidelines of Directive 2010/63/EU. The protocols were approved by the Hamburg Authority for Justice and Consumer Protection, Food and Veterinary Affairs (reference number 100/13) and the UKE Animal Welfare Commission.

### Cell culture

Primary hippocampal cells were isolated from embryonic day 16 (E16) embryos of wild-type C57BL6 and Nbea +/− C57BL6 mice, as previously described^38^, and were seeded onto 12-mm coverslips coated in advance with poly-L-lysine (5 µg/ml in PBS). Neurons were then cultured in vitro (DIV) for 14–18 days at 37 °C in a humified incubator with 5% CO_2_ using Primary Neuron Growth Medium (Lonza, Cat# CC-4461). Cos7 cells derived from African green monkey (LGC Standards, Cat# CRL-1651) were used for live cell imaging and HEK 293 cells were used for biochemical studies. Both were cultured at 37 °C in a humidified incubator with 5% CO_2_ and in Dulbecco’s modified Eagle’s medium (DMEM; LGC Standards, Cat# ATCC-30-2002) supplemented with 10% fetal calf serum FCS, 100 µg/mL streptomycin and 100 units/ml penicillin. For the experiments, 60,000 cells were seeded per 22 mm coverslip. The cells were regularly tested for mycobacteria and other contaminants, but were not additionally authenticated after the first receipt.

### Genotyping

Tail biopsies were taken from 2–4 week-old mice. Genomic DNA was extracted with QuickExtract DNA Extraction Solution (BIOzym, Cat# 101094) and used for PCR with the following primers: WT sense: 5′- GACTAAAAGATGGCAGCTCTC −3′; WT and KO antisense: 5′-TTTCGTACTAGCAAAGGAGTG-3′; KO sense: 5′-TTTGAGCACCAGAGAGGACATC-3′. Subsequently, the presence of the wild-type and knockout alleles was indicated by 595 and 548 bp products, respectively, detected on a 1.5% agarose gel.

### Immunoprecipitation and Pull-down experiments

All steps were performed at 4 °C. Mouse cortices at postnatal day 140 were isolated and placed in IM-Ac buffer (20 mM HEPES, 100 mM K-acetate, 40 mM KCl, 5 mM EGTA, 5 mM MgCl_2_, 1x Complete Protease Inhibitor Cocktail (Sigma-Aldrich, Cat# 4693-13202001), 1 mM PMSF, 5 mM DTT, and 1x PhosSTOP (Sigma-Aldrich, Cat# 4906837001), pH 7.2). Brain lysates were obtained by differential centrifugation as described and dissolved in IM-Ac buffer containing 1% Triton (Saito, Okada et al., 1997). Meanwhile, 30 µL of “Dynabeads Protein G” (Thermo Fisher Scientific, Cat# 10004D) was washed in PBS and incubated with 2–5 µg of the specific antibody or control for 30–60 min. After washing in PBS and IM-Ac buffer, the antibody-coupled beads were incubated with mouse cortical lysate P2 for 2–4 h. The beads were then washed extensively with IM-Ac buffer, boiled in SDS sample buffer and analyzed by Western blotting. Likewise, COS-7 cells were lysed in IM-Ac buffer containing 1% Triton 48 h upon transfection. Lysates were cleaned by differential centrifugation and incubated with GFP-Trap®Magnetic beads (Chromotek, Cat# gntma) to precipitate GFP-coupled proteins. Upon stringent washing with washing buffer (500 mM NaCl, 2 mM EGTA, 5 mM MgCl_2_, 0.1% SDS, 1% Triton X-100and protease inhibitor for three, 15 min each, to remove ancillary proteins, the beads were incubated for 1 h with 4 µg purified gephyrin protein each. Full-length gephyrin was previously expressed in *Escherichia coli* and purified using a two-step protocol as described earlier^60^. After subsequent washing within a size-exclusion buffer (250 mM NaCl, 20 mM Tris-HCl pH 8.0, 1 MM EDTA), the samples were boiled in SDS sample buffer and analyzed by Western blotting.

### Western Blot analysis

Samples were boiled for 5 min at 95 °C in SDS loading buffer and loaded onto a polyacrylamide gel with a gradient of 4–15% to separate the proteins in SDS-PAGE. The proteins were then transferred to polyvinylidene difluoride (PVDF) membranes using a semi-dry blotting system. The membranes were blocked in 5% milk in TBST before being incubated overnight at 4 °C with primary antibodies. After washing and incubation with secondary antibodies coupled to horseradish peroxidase (HRP). The immunoreactive bands were visualized using the chemiluminescence detection system (INTAS Chemo Cam 3.2), and their optical densities were analyzed using ImageJ software (NIH).

### Peptide array-based analysis

Wild-type mouse gephyrin C-domain peptides (GPHN-1 isoform residues 171–375) were purchased from Intavis AG in CelluSpot format. After rinsing the peptide array slides with TBS (50 mM Tris, 150 mM NaCl, pH 7.6) and 0.05% Tween-20 (TBST) for 5 min, the peptide array was incubated with whole brain lysate for 5 h at RT. After extensive washing, the array slides were incubated overnight at 4 °C with anti-neurobeachin antibodies and then with secondary antibodies coupled to horseradish peroxidase (HRP). Chemiluminescence was detected using the Amersham ECL Prime Western Blotting Detection Reagent (GE Healthcare) and the MicroChemi chemiluminescent bio-imaging system (DNR Bio-imaging Systems). The resulting dot blots were analyzed using the array analysis software (Active Motif). The relative affinities and rms deviations shown are from peptide library duplicate analysis.

### Induction of iLTP and GABA stimulation

To induce synaptic changes, DIV 14–16 primary hippocampal neurons were incubated with 10 µM DNQX + 20 µM NMDA (iLTP) or 100 µM GABA or water (negative control) for 3 min at 37 °C each. For recovery, the cells were incubated in fresh medium for 15 min at 37 °C. The cells were then processed as described in the Immunocytochemistry section. For experiments with PKA inhibitor treatment, cells were pretreated with 1 µM KT5720 or 0.4% DMSO (control) before the stimulation protocol was performed in the presence of 1 µM KT5720 or 0.4% DMSO, respectively.

### Antibody feeding assay & live cell staining

DIV 14–16 primary hippocampal neurons were briefly washed with warm timelapse buffer (10 mM HEPES, 135 mM NaCl, 5 mM KCl, 2 mM CaCl_2_, 2 mM MgCl_2_, 15 mM glucose, pH 7.4) and then incubated with primary antibody (anti-GABA_A_Rγ2, 1:100) for 1 h at 4 °C. Cells were then either fixed for live cell staining with 4% paraformaldehyde + 4% sucrose for 5–10 min at room temperature or placed in preconditioned medium plus 100 µM GABA for 30 min at 37 °C. Neurons were fixed with 4% paraformaldehyde + 4% sucrose for 5–10 min at room temperature and used for immunocytochemistry. In a first step the secondary (red) antibody was applied to label cell surface receptors. Afterwards, cells were permeabilized and blocked and the secondary antibody (green) was applied to label internalized receptors. For rescue experiments, transfected neurons were identified through GFP expression.

### Immunocytochemistry

Primary cultured hippocampal neurons from wild-type C57BL6 and Nbea +/− C57BL6 mice were fixed in 4% paraformaldehyde containing 4% sucrose for 5–10 min at room temperature. After fixation, coverslips were washed with PBS and initially stored at 4 °C or immediately used for immunocytochemical staining. Cells were permeabilized and blocked with 10% normal goat serum in PBS + 0.25% Triton X-100 before incubation overnight at 4 °C with primary antibodies. The cells were washed three times in PBS and incubated with secondary antibodies for 1 h. Coverslips were then mounted in Aqua Poly Mount and analyzed using a Nikon microscope equipped with a spinning disk (Yokogawa, Visitron systems), solid-state lasers (488, 561, 647 and 407), objectives (100×, 60x) and two EM-CCD cameras (Hamamatsu Photonics 512/1024×).

### Cloning

GFP-Nbea and mTom-Gephyrin were described previously^38, 61^. Constructs of gephyrin peptides were PCR-amplified from mouse cDNA, tagged with a myc-tag and cloned into pcDNA3.1 using *Hind*III and *Xho*I restriction sites (Invitrogen, Cat#V79020). A ΔPKA construct of Nbea was published previously^37^, and the desired fragment was then cut out using *EcoR*I and *BspT*I to clone it into the GFP-Nbea vector using the same restriction sites. All constructs were verified by dideoxy sequencing.

### Transfections

COS-7 and HEK 293 cells were transfected with TransFectin (BioRAD, Cat# 1703350) according to the manufacturer’s instructions. Cultured hippocampal neurons from *Nbea* +/+ wild-type C57BL6 and *Nbea* +/− C57BL6 mice, regardless of sex, were transfected using a calcium phosphate precipitation protocol. For each coverslip, 2 µg DNA (250 mM CaCl_2_ in 25 µL) was mixed with 25 µL 2× HBS (42 mM HEPES, 10 mM KCl, 12 mM dextrose, 274 mM NaCl, 1.5 mM Na2HPO4; pH 7.0) and added to the culture medium. The precipitates formed were carefully removed after 1 h by washing with timelapse buffer (10 mM HEPES, 135 mM NaCl, 5 mM KCl, 2 mM CaCl_2_, 2 mM MgCl_2_, 15 mM glucose, pH 7.4), and finally 600 µl Primary Neuron Growth Media (Lonza, Cat# CC-4461) was added.

### Live-cell imaging

COS-7 cells were prepared as described above. The coverslips were placed in an Attofluor cell chamber for microscopy (Thermo Fisher Scientific, Cat. No. A-7816) filled with Timelapse buffer (10 mM HEPES, 135 mM NaCl, 5 mM KCl, 2 mM CaCl_2_, 2 mM MgCl_2_, 15 mM glucose, pH 7.4). A Niko microscope equipped with a spinning disk (Yokogawa, Visitron systems), solid-state lasers (488, 561, 647 and 407), objectives (60×), two EM-CCD cameras (Hamamatsu Photonics 512/1024×) and an incubation chamber for a controlled cell culture environment (5% CO_2_ at 37 °C) were used to acquire the images.

### Electrophysiology

Patch-clamp experiments were performed on hippocampal neurons (DIV 11 to 14) in the whole-cell voltage-clamp configuration. Pipettes with a resistance of 3.0–4.0 MΩ were made from borosilicate glass capillaries and then filled with intracellular solution (140 mM CsCl, 1 mM CaCl_2_, 1 mM MgCl_2_, 11 mM EGTA, 5 mM EGTA, 5 mM HEPES, pH adjusted to 7.2 with CsOH). The experiments were then performed at room temperature (21–23 °C) in an extracellular solution (143 mM NaCl, 5 mM KCl, 0.8 mM MgCl_2_, 10 mM HEPES, 5 mM glucose and 0.5 µM TTX, 10 µM CNQX and 50 µM APV, pH adjusted to 7.3 with NaOH). The potential was maintained at −70 mV. Current events were evoked by brief stimulation with 100 µM GABA, followed by recordings for 10 s each. Spontaneous mIPSCs were detected for more than 30 s each after previous stimulation with 100 µM GABA over 4 h. Amplitudes of the evoked potentials were analyzed with Fitmaster, while spikes were detected and analyzed using Clampfit. The different results were compared between *Nbea* +/+ and *Nbea* +/− genotypes, or after treatment with DMSO or PKA inhibitor KT5720 solved in DMSO. Unless otherwise stated, all chemicals were purchased from Sigma.

### Statistical analysis and reproducibility

Unless otherwise stated, three independent technical replicates were performed for each experiment and biological independent samples were then analyzed. Since all data were quantified using automated tools, there was no blinding. Statistical analysis and graphing were performed using GraphPad Prism 9.0.1 (GraphPad Software). During data analysis, outliers were identified using the ROUT method and excluded accordingly. No other inclusion or exclusion criteria were applied. All data were tested for normality using the Shapiro-Wilk test. Either a two-tailed, unpaired Student’s t-test or one-way ANOVA was used to separate the means of normally distributed data, while the Mann-Whitney U test was used to analyze nonparametrically distributed data. Significance was defined as follows: *p < 0.05, **p < 0.01, ***p < 0.001, ****p < 0.0001. Further statistical details on the individual experiments are provided in the respective figure legends.

### Reporting summary

Further information on research design is available in the Nature Portfolio Reporting Summary linked to this article.

## Supplementary information

Supplemental Material

Description of Additional Supplementary Files

SupplementaI Data 1

SupplementaI Data 2

Reporting Summary

## Data Availability

All data generated or analyzed during this study are included in this manuscript. The numerical source data behind the graphs and the corresponding statistical analysis are added as supplementary data 1. The original and unprocessed pictures of the western blots are added as supplementary data 2. Further raw data are available from the corresponding author on reasonable request.

## Code availability

No custom computer code was used within this study and all commercially available programs are listed within the reagent table of the Supplementary Information.

## References

[1] Eichler, S. A. & Meier, J. C. E-I balance and human diseases - from molecules to networking. *Front Mol. Neurosci.* **1**, 2 (2008). 18946535 10.3389/neuro.02.002.2008 PMC2526001

[2] Sohal, V. S. & Rubenstein, J. L. R. Excitation-inhibition balance as a framework for investigating mechanisms in neuropsychiatric disorders. *Mol. Psychiatry* **24**, 1248–1257 (2019). 31089192 10.1038/s41380-019-0426-0 PMC6742424

[3] Jacob, T. C., Moss, S. J. & Jurd, R. GABA(A) receptor trafficking and its role in the dynamic modulation of neuronal inhibition. *Nat. Rev. Neurosci.* **9**, 331–343 (2008). 18382465 10.1038/nrn2370 PMC2709246

[4] Kittler, J. T. et al. Regulation of synaptic inhibition by phospho-dependent binding of the AP2 complex to a YECL motif in the GABAA receptor gamma2 subunit. *Proc. Natl Acad. Sci. USA* **105**, 3616–3621 (2008). 18305175 10.1073/pnas.0707920105 PMC2265186

[5] Ali, H., Marth, & Krueger-Burg, D. Neuroligin-2 as a central organizer of inhibitory synapses in health and disease. *Sci Signal*, **13** (2020). 10.1126/scisignal.abd8379 33443230

[6] Soykan, T. et al. A conformational switch in collybistin determines the differentiation of inhibitory postsynapses. *EMBO J.* **33**, 2113–2133 (2014). 25082542 10.15252/embj.201488143 PMC4195776

[7] Kneussel, M. & Betz, H. Clustering of inhibitory neurotransmitter receptors at developing postsynaptic sites: the membrane activation model. *Trends Neurosci.* **23**, 429–435 (2000). 10941193 10.1016/s0166-2236(00)01627-1

[8] Sola, M. et al. X-ray crystal structure of the trimeric N-terminal domain of gephyrin. *J. Biol. Chem.* **276**, 25294–25301 (2001). 11325967 10.1074/jbc.M101923200

[9] Dos Reis, R. et al. Complex regulation of Gephyrin splicing is a determinant of inhibitory postsynaptic diversity. *Nat. Commun.* **13**, 3507 (2022). 35717442 10.1038/s41467-022-31264-w PMC9206673

[10] Poulopoulos, A. et al. Neuroligin 2 drives postsynaptic assembly at perisomatic inhibitory synapses through gephyrin and collybistin. *Neuron* **63**, 628–642 (2009). 19755106 10.1016/j.neuron.2009.08.023

[11] Mayer, S. et al. Collybistin activation by GTP-TC10 enhances postsynaptic gephyrin clustering and hippocampal GABAergic neurotransmission. *Proc. Natl. Acad. Sci. USA* **110**, 20795–20800 (2013). 24297911 10.1073/pnas.1309078110 PMC3870750

[12] Kneussel, M. et al. Loss of postsynaptic GABA(A) receptor clustering in gephyrin-deficient mice. *J. Neurosci.* **19**, 9289–9297 (1999). 10531433 10.1523/JNEUROSCI.19-21-09289.1999 PMC6782938

[13] Essrich, C. et al. Postsynaptic clustering of major GABAA receptor subtypes requires the gamma 2 subunit and gephyrin. *Nat. Neurosci.* **1**, 563–571 (1998). 10196563 10.1038/2798

[14] Maric, H. M. et al. Molecular basis of the alternative recruitment of GABA(A) versus glycine receptors through gephyrin. *Nat. Commun.* **5**, 5767 (2014). 25531214 10.1038/ncomms6767

[15] Zacchi, P., Antonelli, R. & Cherubini, E. Gephyrin phosphorylation in the functional organization and plasticity of GABAergic synapses. *Front Cell Neurosci.* **8**, 103 (2014). 24782709 10.3389/fncel.2014.00103 PMC3988358

[16] Comenencia-Ortiz, E., Moss, S. J. & Davies, P. A. Phosphorylation of GABAA receptors influences receptor trafficking and neurosteroid actions. *Psychopharmacol. (Berl.)* **231**, 3453–3465 (2014). 10.1007/s00213-014-3617-z PMC4135009 24847959

[17] Niwa, F. et al. cAMP-EPAC-Dependent Regulation of Gephyrin Phosphorylation and GABA(A)R Trapping at Inhibitory Synapses. *iScience* **22**, 453–465 (2019). 31835170 10.1016/j.isci.2019.11.013 PMC6926171

[18] Tyagarajan, S. K. & Fritschy, J. M. Gephyrin: a master regulator of neuronal function? *Nat. Rev. Neurosci.* **15**, 141–156 (2014). 24552784 10.1038/nrn3670

[19] Halff, E. F. et al. Phosphorylation of neuroligin-2 by PKA regulates its cell surface abundance and synaptic stabilization. *Sci. Signal* **15**, eabg2505 (2022). 35727864 10.1126/scisignal.abg2505

[20] Petrini, E. M. et al. Synaptic recruitment of gephyrin regulates surface GABAA receptor dynamics for the expression of inhibitory LTP. *Nat. Commun.* **5**, 3921 (2014). 24894704 10.1038/ncomms4921 PMC4059940

[21] Tehrani, M. H. & Barnes, E. M. Jr., Agonist-dependent internalization of gamma-aminobutyric acidA/benzodiazepine receptors in chick cortical neurons. *J. Neurochem.* **57**, 1307–1312 (1991). 1654391 10.1111/j.1471-4159.1991.tb08295.x

[22] Kittler, J. T. et al. Constitutive endocytosis of GABAA receptors by an association with the adaptin AP2 complex modulates inhibitory synaptic currents in hippocampal neurons. *J. Neurosci.* **20**, 7972–7977 (2000). 11050117 10.1523/JNEUROSCI.20-21-07972.2000 PMC6772725

[23] Kittler, J. T. et al. Phospho-dependent binding of the clathrin AP2 adaptor complex to GABAA receptors regulates the efficacy of inhibitory synaptic transmission. *Proc. Natl. Acad. Sci. USA* **102**, 14871–14876 (2005). 16192353 10.1073/pnas.0506653102 PMC1253579

[24] Nakamura, Y. et al. Phosphorylation on Ser-359 of the alpha2 subunit in GABA type A receptors down-regulates their density at inhibitory synapses. *J. Biol. Chem.* **295**, 12330–12342 (2020). 32620552 10.1074/jbc.RA120.014303 PMC7458806

[25] Antonelli, R. et al. Pin1-dependent signalling negatively affects GABAergic transmission by modulating neuroligin2/gephyrin interaction. *Nat. Commun.* **5**, 5066 (2014). 25297980 10.1038/ncomms6066 PMC4197815

[26] Brandon, N. J. et al. A-kinase anchoring protein 79/150 facilitates the phosphorylation of GABA(A) receptors by cAMP-dependent protein kinase via selective interaction with receptor beta subunits. *Mol. Cell Neurosci.* **22**, 87–97 (2003). 12595241 10.1016/s1044-7431(02)00017-9

[27] Snyder, E. M. et al. Role for A kinase-anchoring proteins (AKAPS) in glutamate receptor trafficking and long term synaptic depression. *J. Biol. Chem.* **280**, 16962–16968 (2005). 15718245 10.1074/jbc.M409693200 PMC3923403

[28] Wang, X. et al. Neurobeachin: A protein kinase A-anchoring, beige/Chediak-higashi protein homolog implicated in neuronal membrane traffic. *J. Neurosci.* **20**, 8551–8565 (2000). 11102458 10.1523/JNEUROSCI.20-23-08551.2000 PMC6773050

[29] Volders, K., Nuytens, K. & Creemers, J. W. The autism candidate gene Neurobeachin encodes a scaffolding protein implicated in membrane trafficking and signaling. *Curr. Mol. Med.* **11**, 204–217 (2011). 21375492 10.2174/156652411795243432

[30] Su, Y. et al. Neurobeachin is essential for neuromuscular synaptic transmission. *J. Neurosci.* **24**, 3627–3636 (2004). 15071111 10.1523/JNEUROSCI.4644-03.2004 PMC6729756

[31] Niesmann, K. et al. Dendritic spine formation and synaptic function require neurobeachin. *Nat. Commun.* **2**, 557 (2011). 22109531 10.1038/ncomms1565 PMC3482631

[32] Nair, R. et al. Neurobeachin regulates neurotransmitter receptor trafficking to synapses. *J. Cell Biol.* **200**, 61–80 (2013). 23277425 10.1083/jcb.201207113 PMC3542797

[33] Castermans, D. et al. The neurobeachin gene is disrupted by a translocation in a patient with idiopathic autism. *J. Med. Genet.* **40**, 352–356 (2003). 12746398 10.1136/jmg.40.5.352 PMC1735479

[34] Mulhern, M. S. et al. NBEA: Developmental disease gene with early generalized epilepsy phenotypes. *Ann. Neurol.* **84**, 788–795 (2018). 30269351 10.1002/ana.25350 PMC6249120

[35] Nuytens, K. et al. Haploinsufficiency of the autism candidate gene Neurobeachin induces autism-like behaviors and affects cellular and molecular processes of synaptic plasticity in mice. *Neurobiol. Dis.* **51**, 144–151 (2013). 23153818 10.1016/j.nbd.2012.11.004

[36] Odent, P. et al. Spectrum of social alterations in the Neurobeachin haploinsufficiency mouse model of autism. *Brain Res. Bull.* **167**, 11–21 (2021). 33197534 10.1016/j.brainresbull.2020.11.007

[37] Farzana, F. et al. Neurobeachin regulates glutamate- and GABA-Receptor targeting to synapses via distinct pathways. *Mol. Neurobiol.* **53**, 2112–2123 (2016). 25934101 10.1007/s12035-015-9164-8 PMC4823379

[38] Gromova, K. V. et al. Neurobeachin and the kinesin KIF21B are critical for endocytic recycling of NMDA receptors and regulate social behavior. *Cell Rep.* **23**, 2705–2717 (2018). 29847800 10.1016/j.celrep.2018.04.112

[39] Jacob, T. C. et al. Gephyrin regulates the cell surface dynamics of synaptic GABAA receptors. *J. Neurosci.* **25**, 10469–10478 (2005). 16280585 10.1523/JNEUROSCI.2267-05.2005 PMC6725824

[40] Muellerleile, J. et al. Enhanced LTP of population spikes in the dentate gyrus of mice haploinsufficient for neurobeachin. *Sci. Rep.* **10**, 16058 (2020). 32994505 10.1038/s41598-020-72925-4 PMC7524738

[41] Nguyen, P. V. & Woo, N. H. Regulation of hippocampal synaptic plasticity by cyclic AMP-dependent protein kinases. *Prog. Neurobiol.* **71**, 401–437 (2003). 15013227 10.1016/j.pneurobio.2003.12.003

[42] Hernandez-Vivanco, A. et al. Protein kinase a-dependent plasticity of local inhibitory synapses from hilar somatostatin-expressing neurons. *eNeuro* **10** (2023). 10.1523/ENEURO.0089-23.2023 PMC10561540 37734950

[43] del Pino, I. et al. The trafficking proteins Vacuolar Protein Sorting 35 and Neurobeachin interact with the glycine receptor beta-subunit. *Biochem. Biophys. Res. Commun.* **412**, 435–440 (2011). 21821005 10.1016/j.bbrc.2011.07.110

[44] Zita, M. M. et al. Post-phosphorylation prolyl isomerisation of gephyrin represents a mechanism to modulate glycine receptors function. *EMBO J.* **26**, 1761–1771 (2007). 17347650 10.1038/sj.emboj.7601625 PMC1847658

[45] Groeneweg, F. L. et al. Gephyrin: a key regulatory protein of inhibitory synapses and beyond. *Histochem Cell Biol.* **150**, 489–508 (2018). 30264265 10.1007/s00418-018-1725-2

[46] Miyamoto, E., Kuo, J. F. & Greengard, P. Adenosine 3’,5’-monophosphate-dependent protein kinase from brain. *Science* **165**, 63–65 (1968). 4306869

[47] Bourne, H. R., Tomkins, G. M. & Dion, S. Regulation of phosphodiesterase synthesis: requirement for cyclic adenosine monophosphate-dependent protein kinase. *Science* **181**, 952–954 (1973). 4354229 10.1126/science.181.4103.952

[48] Karbon, E. W., Duman, R. S. & Enna, S. J. GABAB receptors and norepinephrine-stimulated cAMP production in rat brain cortex. *Brain Res.* **306**, 327–332 (1984). 6087977 10.1016/0006-8993(84)90382-2

[49] Luscher, B., Fuchs, T. & Kilpatrick, C. L. GABAA receptor trafficking-mediated plasticity of inhibitory synapses. *Neuron* **70**, 385–409 (2011). 21555068 10.1016/j.neuron.2011.03.024 PMC3093971

[50] Tyagarajan, S. K. et al. Extracellular signal-regulated kinase and glycogen synthase kinase 3beta regulate gephyrin postsynaptic aggregation and GABAergic synaptic function in a calpain-dependent mechanism. *J. Biol. Chem.* **288**, 9634–9647 (2013). 23408424 10.1074/jbc.M112.442616 PMC3617267

[51] Flores, C. E. et al. Activity-dependent inhibitory synapse remodeling through gephyrin phosphorylation. *Proc. Natl. Acad. Sci. USA* **112**, E65–E72 (2015). 25535349 10.1073/pnas.1411170112 PMC4291629

[52] Smith, K. R. et al. Stabilization of GABA(A) receptors at endocytic zones is mediated by an AP2 binding motif within the GABA(A) receptor beta3 subunit. *J. Neurosci.* **32**, 2485–2498 (2012). 22396422 10.1523/JNEUROSCI.1622-11.2011 PMC6621817

[53] Lombardi, J. P., Kinzlmaier, D. A. & Jacob, T. C. Visualizing GABA A receptor trafficking dynamics with fluorogenic protein labeling. *Curr. Protoc. Neurosci.* **92**, e97 (2020). 32364672 10.1002/cpns.97 PMC7556711

[54] Jurd, R. & Moss, S. J. Impaired GABA(A) receptor endocytosis and its correlation to spatial memory deficits. *Commun. Integr. Biol.* **3**, 176–178 (2010). 20585515 10.4161/cib.3.2.10742 PMC2889979

[55] Ji, M. H. et al. Overinhibition mediated by parvalbumin interneurons might contribute to depression-like behavior and working memory impairment induced by lipopolysaccharide challenge. *Behav. Brain Res.* **383**, 112509 (2020). 31987933 10.1016/j.bbr.2020.112509

[56] Terunuma, M. et al. Postsynaptic GABAB receptor activity regulates excitatory neuronal architecture and spatial memory. *J. Neurosci.* **34**, 804–816 (2014). 24431439 10.1523/JNEUROSCI.3320-13.2013 PMC3891960

[57] Asaka, Y. et al. Hippocampal synaptic plasticity is impaired in the Mecp2-null mouse model of Rett syndrome. *Neurobiol. Dis.* **21**, 217–227 (2006). 16087343 10.1016/j.nbd.2005.07.005

[58] Tretter, V. et al. Deficits in spatial memory correlate with modified gamma-aminobutyric acid type A receptor tyrosine phosphorylation in the hippocampus. *Proc. Natl. Acad. Sci. USA* **106**, 20039–20044 (2009). 19903874 10.1073/pnas.0908840106 PMC2785288

[59] Medrihan, L. et al. Neurobeachin a protein implicated in membrane protein traffic and autism is required for the formation and functioning of central synapses. *J. Physiol*. **587**, 5095–5106 (2009). 10.1113/jphysiol.2009.178236 PMC2790251 19723784

[60] Kim, E. Y. et al. Deciphering the structural framework of glycine receptor anchoring by gephyrin. *EMBO J.* **25**, 1385–1395 (2006). 16511563 10.1038/sj.emboj.7601029 PMC1422172

[61] Rathgeber, L. et al. GSK3 and KIF5 regulate activity-dependent sorting of gephyrin between axons and dendrites. *Eur. J. Cell Biol.* **94**, 173–178 (2015). 10.1016/j.ejcb.2015.01.005 25701174

