# Supplementary material for: Neurobeachin regulates receptor downscaling at GABAergic inhibitory synapses in a protein kinase A-dependent manner
Source: Commun Biol. 2024 Dec 12;7:1635. doi: 10.1038/s42003-024-07294-z (PMC11638247; doi:10.1038/s42003-024-07294-z)
Supplement: Supplementary file 1 — Supplemental Material [file 42003_2024_7294_MOESM1_ESM.pdf]

SUPPLEMENTARY INFORMATION

Neurobeachin regulates receptor downscaling at GABAergic inhibitory synapses in a protein kinase A-dependent manner

Felix P. Lützenkirchen[1], Yipeng Zhu[1], Hans M. Maric[3], Dominik S. Boeck[1], Kira V. Gromova[1*], Matthias Kneussel[1,2*]

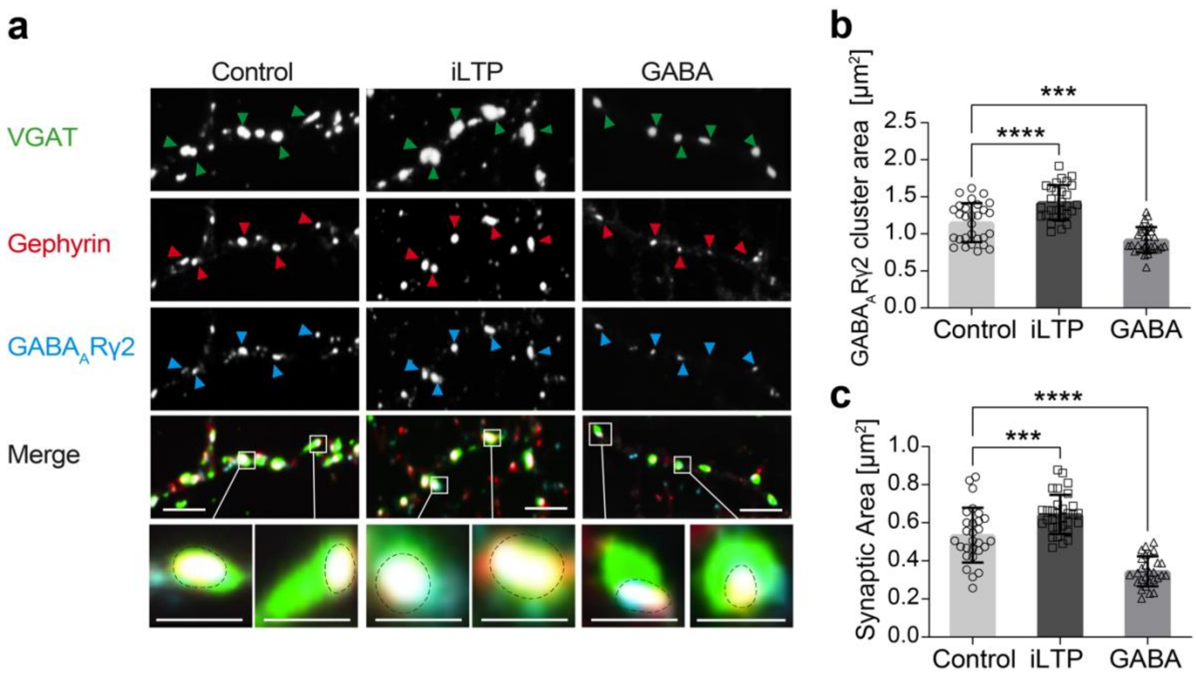

Supplementary Fig. 1 (related to Fig.1). Chemically induced plasticity protocols alter GABA-ergic synapses. (**a**) Hippocampal neurons from Nbea +/+ treated with a CNQX/NMDA protocol to induce iLTP or GABA were coimmunostained with specific antibodies for endogenous VGAT (green), endogenous gephyrin (red) and surface GABA$_A$Rγ2 (cyan). The arrows show examples of colocalized signals. (**b**) Average GABA$_A$Rγ2 cluster area (µm^2) measured and compared after iLTP (n=31), GABA stimulation (n=28) or control (n=28) as in a (control vs. iLTP ****p=0.0001; control vs. GABA ***p=0. 0004). (**c**) Synaptic area determined by colocalization of VGAT, GABA$_A$Rγ2 and gephyrin after iLTP (n=31), GABA stimulation (n=28) or control (n=28) as in a (control vs. iLTP ***p=0.0006; control vs. GABA ****p<0.0001). Scale bars: 2µm. The data are given as mean ± SD. Statistical significance was determined using a standard one-way ANOVA (b,c).

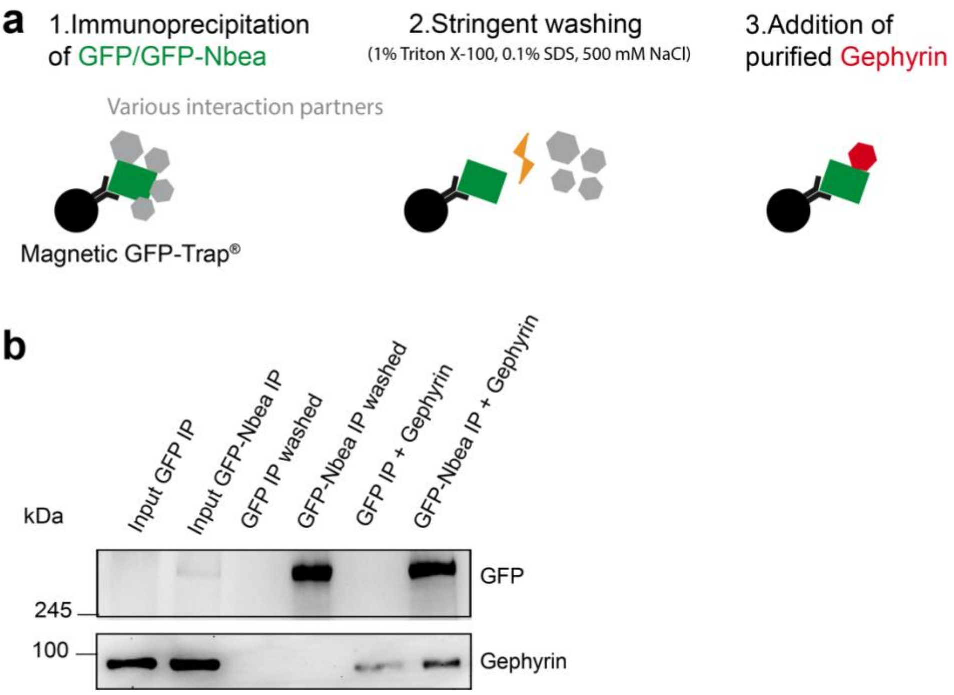

Supplementary Fig. 2 (related to Fig. 2). GFP-Nbea pulls down purified gephyrin. (**a**) Schematic representation of the pull-down assay: (1) Immunoprecipitation of GFP or GFP-Nbea (green) and the different interaction partners (gray) from fibroblast-like COS-7 cells using Magnetic GFP-Trap®. (2) Stringent washing of the magnetic beads with a buffer containing 1% Triton X-100, 0. 1% SDS, 500 mM NaCl to remove various interaction partners. (3) Addition of purified gephyrin to the GFP- / GFP-Nbea-coupled beads (**b**) Immunodetection of GFP and gephyrin in the different steps of the pull-down.

a

	1	2	3	4	5	6	7	8	9	10	11	12	13	14	15	16	17	18	19	20	21	22	23	24
A	Gly B 1	Gly B 2	Gly B 3	Gly B 4	Gly B 5	Gly B 6	Gly B 7	Gly B 8	Gly B 9	Gly B 10	Gly B 11	Gly B 12	Gly B 13	Gly B 14	Gly B 15	Gly B 16	Gly B 17	GPH 1	GPH 2	GPH 3	GPH 4	GPH 5	GPH 6	GPH 7
B	GPH 8	GPH 9	GPH 10	GPH 11	GPH 12	GPH 13	GPH 14	GPH 15	GPH 16	GPH 17	GPH 18	GPH 19	GPH 20	GPH 21	GPH 22	GPH 23	GPH 24	GPH 25	GPH 26	GPH 27	GPH 28	GPH 29	GPH 30	GPH 31
C	GPH 32	GPH 33	GPH 34	GPH 35	GPH 36	GPH 37	GPH 38	GPH 39	NL 2 1	NL 2 2	NL 2 3	NL 2 4	NL 2 5	NL 2 6	NL 2 7	NL 2 8	NL 2 9	NL 2 10	NL 2 11	NL 2 12	NL 2 13	NL 2 14	NL 2 15	NL 2 16
D	NL 2 17	NL 2 18	NL 2 19	NL 2 20	NL 2 21	NL 2 22	NL 2 23	NL 2 24	NL 2 25	NL 4 1	NL 4 2	NL 4 3	NL 4 4	NL 4 5	NL 4 6	NL 4 7	NL 4 8	NL 4 9	NL 4 10	NL 4 11	NL 4 12	NL 4 13	NL 4 14	NL 4 15
E	NL 4 16	NL 4 17	NL 4 18	NL 4 19	NL 4 20	NL 4 21	NL 4 22	NL 4 23	NL 4 24	NL 4 25	NL 4 26	NL 4 27	NL 4 28	NL 4 29	NL 4 30	NL 4 31	CB YT 1	CB YT 2	CB YT 3	CB3 1	CB3 2	CB3 3	Mkl 1	Mkl 2

b

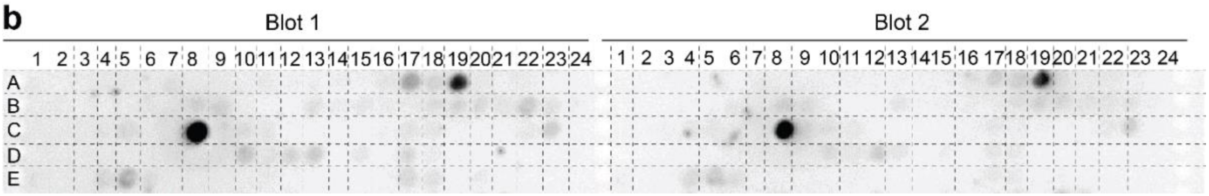

Supplementary Fig. 3 (related to Fig. 3). Nbea is detected with gephyrin peptides. (a) Reading frame for the peptide array blot with different peptides of glycine receptor beta (Gly B), gephyrin (GPH), neuroligin 2 (NL 2), neuroligin 4 (NL 4), control blank (CB YT), (CB3), (Mkl) (b) Immunodetection of Nbea on the peptide array blot 1 (left) and blot 2 (right) according to the blot grid in a.

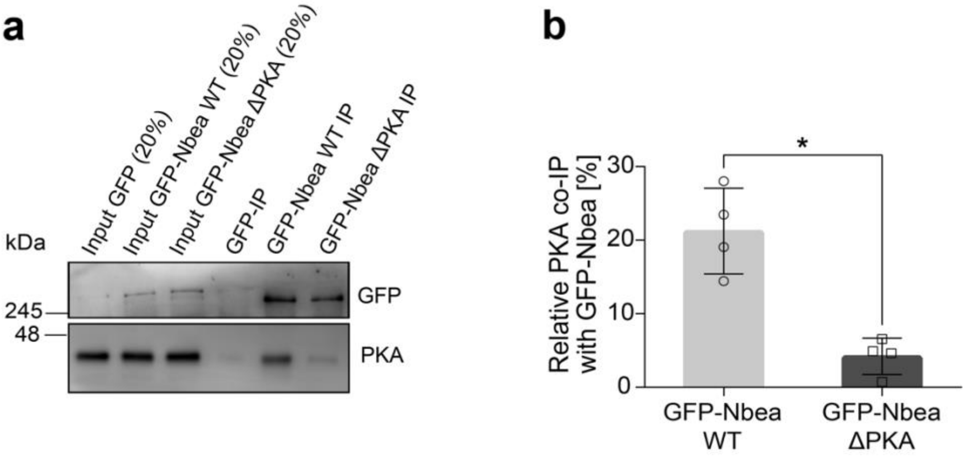

Supplementary Fig. 4 (related to Fig. 7). Decreased binding of PKA to NbeaΔPKA. (**a**) Immunoprecipitation of GFP from HEK 293 cells expressing GFP, GFP-Nbea WT or GFP-Nbea ΔPKA and co-immunoprecipitation of PKA. (**b**) Quantification of the intensity of PKA relative to GFP-Nbea after expression and immunoprecipitation of GFP-Nbea WT or GFP-Nbea ΔPKA (*p=0.0137) N=4 independent experiments. Data expressed as mean ± SD. Statistical significance determined by paired Student t-test (b).

Supplementary Table 1: Reagent used in this study

Reagent or Resource	Source	Identifier
**Antibodies**		
Guinea Pig anti-GABA$_A$R-γ2 (imaging)	Synaptic system	Cat# 224 004, RRID: AB_10594245
Rabbit anti-GABA$_A$R-γ2 (WB, IP)	Synaptic systems	Cat# 224 003, RRID: AB_2263066
Mouse anti-Gephyrin (3B11; imaging, WB, IP)	Synaptic systems	Cat# 147 111, RRID: AB_2619837
Mouse anti-PKA[C] (WB, IP)	BD Biosciences	Cat# 610980, RRID: AB_398293
Mouse anti-myc (imaging)	MilliporeSigma	Cat# M4439, RRID: AB_10888756
Rabbit anti-Neurobeachin	Synaptic systems	Cat# 194 003, RRID: AB_2149112
Guinea Pig anti-VGAT	Synaptic systems	Cat# 131 004, RRID: AB_887873
Rabbit anti-VGAT	Synaptic systems	Cat# 131 003, RRID: AB_887867
IRDye® 800CW Goat Anti-mouse IgG	LI-COR Biosciences	Cat# 926-32210; RRID: AB_621842
IRDye® 800CW Goat Anti-rabbit IgG	LI-COR Biosciences	Cat# 926-32211; RRID: AB_621843
Donkey anti-rabbit Alexa 488 conjugated	Jackson Immuno	Cat# 711-546-152, RRID: AB_2340619
Donkey anti-mouse LC HRP conjugated	Jackson Immuno	Cat# 715-036-151, RRID: AB_2340774
Donkey anti-rabbit LC HRP conjugated	Jackson Immuno	Cat# 715-036-152, RRID: AB_2340774
Donkey anti-mouse Cy3 conjugated	Jackson Immuno	Cat# 715-165-150, RRID: AB_2340813
Donkey anti-mouse Cy5 conjugated	Jackson Immuno	Cat# 715-175-150, RRID:AB_2340819
Donkey anti-guinea pig Cy5 conjugated	Jackson Immuno	Cat# 706-175-148, RRID: AB_2340477
Donkey anti-guinea pig Cy3 conjugated	Jackson Immuno	Cat# 706-165-148, RRID: AB_2340474
Donkey anti-guinea pig Alexa 488 conjugated	Jackson Immuno	Cat# 706-545-148, RRID: AB_2340472
**Chemicals, peptides, recombinant proteins**		
γ-Aminobutyric acid	Sigma-Aldrich	Cat# 56-12-2
DNQX	Sigma-Aldrich	Cat# 2379-57-9
N-Methyl-D-aspartic acid	Sigma-Aldrich	Cat# 6384-92-5
DMSO	Sigma-Aldrich	Cat# 67-68-5
Complete protease inhibitor tablets	Sigma-Aldrich	Cat# 4693132001
PhosSTOP^{TM}	Sigma-Aldrich	Cat# 4906837001
KT5720	Cayman Chemical	Cat# 10011011-50
Aqua-PolyMount	Polysciences	Cat# 18606
Dynabeads Protein G	Thermo Fisher	Cat# 10004D
GFP-Trap® Magnetic beads	Chromotek	Cat# gntma
TransFectin^{TM}	Bio-Rad	Cat# 1703350

VECTASTAIN Elite ABC-HRP Kit	Vector Laboratories	Cat# PK-6100
Primary Neuron Growth Media bulletkit	Lonza	Cat# 7278
Dulbecco's Modified Eagle's Medium	LGC Standards	Cat# ATCC-30-2002
QuickExtract DNA Extraction	BIOzym	Cat# 101094
**Cell lines**		
COS-7	LGC Standards	Cat# CRL-1651; RRID: CVCL_0224
HEK 293	LGC Standards	Cat# CRL-1573; RRID: CVCL_R293
Mouse: Nbea	[60]	N/A
**Oligonucleotides**		
WT sense: 5′CCCTGGTGTTCCTTTCTTCTC3′	Eurofins Genomics	N/A
WT and KO antisense: 5′AAACCTGGGCAAAGGCATAC3′	Eurofins Genomics	N/A
KO sense: 5′GAAATGACCGACCAAGCGACG3′	Eurofins Genomics	N/A
Peptides-forward: 5'TAAAAAAAGCTTACCATGGAACAAAAAC3'	Invitrogen	N/A
Peptide 2-antisense: 5'TATTTCTCGAGTTATGGTGGGGAAG3'	Invitrogen	N/A
Peptide 39-antisense: 5'TATTTCTCGAGTTATGTACCAAGC3'		
Peptide 19-antisense: 5'TATTTCTCGAGTTAAGCCTGGG3'	Invitrogen	N/A
**Plasmids**		
GFP-Nbea	[38]	N/A
mTom-Gephyrin	[61]	N/A
pcDNA-3.1 myc- VKEVHDELEDLPSPP	here	N/A
pcDNA-3.1 myc- KAFITVLEMTPVLGT	here	N/A
pcDNA-3.1 myc- ASLSTTPSESPRAQA	here	N/A
ptdmTom-C1	Clonetech/ TaKaRa	Cat# 632533
pEGFP-C2	Clonetech/TaKaRa	Cat# 632522
GFP Nbea dPKA	[37]	N/A
**Software and algorithms**		
Fiji	NIH	http://fijii.sc
ImageJ 1.38	NIH	https://ImageJ.nih.gov
Prism 9.0.1	GraphPad Software	https://www.graphpad.com
Clampfit	Molecular Devices	https://moleculardevices.com
Fitmaster 2.69	HEKA	https://www.heka.com
