# Supplementary material for: Neurobeachin regulates receptor downscaling at GABAergic inhibitory synapses in a protein kinase A-dependent manner
Source: Commun Biol. 2024 Dec 12;7:1635. doi: 10.1038/s42003-024-07294-z (PMC11638247; doi:10.1038/s42003-024-07294-z)
Supplement: Supplementary file 2 — Description of Additional Supplementary Files [file 42003_2024_7294_MOESM2_ESM.pdf]

Description of Additional Supplementary Files

File name: Supplementary Data 1

Description: The numerical source data behind the graphs in the paper and their statistical analysis.

File name: Supplementary Data 2

Description: The original pictures behind the western blots in the paper.
